# Antifertility activity of methanolic bark extract of *Aegle marmelos* (l.) in male wistar rats

**DOI:** 10.1186/2008-2231-20-94

**Published:** 2012-12-13

**Authors:** Shyam S Agrawal, Ashok Kumar, Sumeet Gullaiya, Vishal Dubey, Ashish Nagar, Poonam Tiwari, Parul Dhar, Varun Singh

**Affiliations:** 1 Delhi Institute of Pharmaceutical Sciences and Research (DIPSAR), Pushp Vihar, Sector III, M. B. Road, New Delhi, 110017, India; 2 Innovative College of Pharmacy, Plot No. 6, Knowledge Park II, Greater Noida, Gautam Buddha Nagar, Uttar Pradesh, India; 3 College of Basic Sciences and Humanities, Punjab Agricultural University, Ludhiana, Punjab, India

**Keywords:** *Aegle marmelos*, Acrosomal, Infertility, Sperm

## Abstract

**Background:**

*Aegle marmelos* leaf, seed and fruit from earlier studies is known to affect male fertility in reversible manner. However they had delayed onset and recovery was found to be prolonged. The present study was undertaken with an aim to evaluate the effect of *Aegle marmelos* bark extract on rats as the extract is found to be a rich source of marmin and fagarine known for reducing male fertility. Three different concentration of methanolic bark extracts of *Aegle marmelos* (L.) were evaluated for male antifertility activity on albino wistar rats. Methanolic bark extract of *Aegle marmelos* at the dose of 200, 400, and 600 mg/Kg b.w was administered orally for 60 days. Treatments were stopped thereafter and animals were sacrificed after a recovery period of 30 days. Control animal were administered vehicle (0.5% CMC for 60 days). Lonidamine was used as standard drug to compare the effect of extract.

**Results:**

Methanolic extract causes a dose & duration dependent infertility via reducing reproductive organ weight and serum testosterone levels. Sperm analysis results showed reduction in sperm density, motility, viability and sperm acrosomal integrity without interfering libido and vital organ body weight. Histopathological studies of testes revealed exfoliation of elongated spermatids, nuclear chromatin condensation, degeneration and prominent spaces detected within the germinal epithelium signifying testicular cytotoxicity and necrosis. Time dependent complete infertility was observed in all dose levels. Animals after the withdrawal from treatment, for 30 days showed restoration of the morphological as well as physiological parameters in extract treated rats. Methanolic extract showed lipid lowering activity compared to control, suggestive good candidature of this plant for further studies.

**Conclusions:**

Our studies suggested *Aegle marmelos* barks methanolic extract as strong candidate for male contraceptive via its ability to produce complete inhibition of pregnancy, rapid restoration of fertility after withdrawal from treatment and its lipid correcting ability proving further beneficial effects.

## Introduction

Higher use of contraceptive methods is a direct indicator of health, population, development and women's empowerment. It also serves as a proxy measure to access the reproductive health services that are essential for meeting many of the millennium development goals, especially for those concerned with child mortality, maternal health, HIV/AIDS, and gender equality [1]. The female contraceptive methods were always on higher priority and men willing to share in the responsibilities of family planning have fewer options of contraceptions which are effective, reversible, non irritating and highly expectable. A survey conducted by WHO showed that 61% of the males using contraceptive were motivated by problems of the female partner, including 35% who had experienced a contraceptive failure, or by the desire to share responsibility [2]. Around 80% of men and their partners wanted to continue, with the male method regimen [2]. Various scientists across the world are constantly trying to develop a reversible, effective and patient compliant male contraceptive. Development of male spermicidal is an effective method for regulating male fertility. Earlier studies from our lab has already demonstrated post coital pre implantation contraceptive ability of H_2_ receptor blocker [3,4] and chroman derivative (isolated from herb) [5]. Now, whether contraception achieved by these agents are limited to females or whether they posses some activity on male reproductive system, these herbal derivatives were evaluated for male contraception. We worked ahead in the field of male contraceptive especially with commonly available herbal and synthetic compounds which can be easily accepted by the end users. There are several medicinal plants associated with male antifertility potential [6]. *Aegle marmelos* (L.) Corr. (Rutaceae) commonly known as the bael fruit tree [7], is used since ages for treatment of numerous diseases [8]. Bioactive compounds isolated from this wonderful plant includes skimmianine, aegeline, lupeol, cineol, citral, citronella, cumin aldehyde, eugenol, marmesinine [9] and are being used for treatment of asthma, anemia, fractures, wounds healing, swollen joints, high blood pressure, jaundice & diarrhoea [10]. Till date, no specific reports are available on the possible male contraceptive properties of *Aegle marmelos* bark extract, however aqueous and ethanolic extract of *Aegle marmelos* leaf, seed and fruit form earlier studies is known to affect male fertility by reducing sperm motility [11-13] and antitesticular [14] activity in reversible manner however their recovery was found to be prolonged [11]. A pilot studies shows methanolic extract was more potent than aqueous and ethanolic extract.

## Materials and methods

### Collection and authentication of crude drug

Bark of *Aegle marmelos* were collected from local market of Delhi, India in the month of September, 2010 and were identified by botanist Dr. H. B. Singh, HOD, Dept. of Botany at National Institute of Science Communication and Information Resources (NISCAIR) on 21^st^ September, 2010 reference no NISCAIR/RHMDC/Consult/2010-11/1536/134, New Delhi, India.

### Preparation of methanol extract

The bark (5.0 kg) was shade dried and pulverized in an electric grinder (Bajaj Bravo3, India); 2.0 kg of the powdered drug was extracted with 6.0 L of methanol for 48 h with occasional shaking. The filtrate (2.0 L) was concentrated under reduced pressure at 40°C to yield 60 g (6.0% w/w) of a brown soluble residue [15]. The residue was further dried in an oven at 37°C to eliminate traces of methanol solvent and stored in a sealed dark airtight plastic container at 4–8°C, until use. The crude residue suspended in (0.5% CMC w/v) served as the doses form for experimentation.

### Experimental design

Male albino wistar rats (200–250 g), procured from the animal house of Delhi Institute of Pharmaceutical Sciences and Research (DIPSAR), New Delhi, were used. Rats were housed in temperature controlled room; 25 ± 2°C with a 12-hour light/dark cycle and 57 ± 7% relative humidity under standard hygienic conditions and had free access to fresh tap water & pelleted diet. The animals were acclimatized for seven days prior to experimental use. The study was done with prior approval from institutional animal ethical committee (IAEC) vide protocol no. (IAEC/DIPSAR/20010-I/15) DIPSAR, New Delhi.

### Treatment phase

The animals were divided into five groups: Control group (n = 6 animals), Standard Drug (SD) 50 mg/rat/day/p.o Lonidamine [16] (n = 18 animals). Group I (G I), Group II (G II), Group III (G III), (n = 24 animals each), The animals that served as control, were treated with 2.0 ml of 0.5 percent CMC each day for 60 days G I, G II, G III were give methanolic bark extract at a dose of 200, 400 and 600 mg/kg b.w respectively. Dose selection was based upon earlier studies done on this plant extract to perform anti diabetic activity at a safe dose levels of 600 mg/kg as no reported LD50 on bark extract were available [15]. Six animals were sacrificed from SD, G I, G II, and G III groups after 24 hours of 20^th^, 40^th^ and 60^th^ days of treatment.

### Recovery phase

After 60 days of the treatment schedule of methanolic bark extract at a dose of 200, 400 and 600 mg/kg b.w of RG I, II & III respectively (n = 6) were withdrawn from treatment for next 30 days. Animals were sacrificed and recovery was assessed.

### Safety evaluation

Blood samples were collected by cardiac puncture and were used for routine hematology and serum clinical chemistry to evaluate the effect of treatment on the other body function and toxicity, if any.

### Hematology

Hematological parameters like red blood cell, pack cell volume, total leukocyte, & hemoglobin were done with the help of Coulter ACT diff (Beckman coulter India Pvt. ltd, Mumbai). erythrocyte sedimentation rate (ESR) done by wintrobe method.

### Clinical chemistry

Serum glutamate oxalate transaminase (SGOT), serum glutamate pyruvate transaminase (SGPT), Creatinine (CR), Blood Urea Nitrogen (BUN), Uric acid (UA) alkaline phosphatase (ALP), Cholesterol, Total Protein (TP) and Albumin (ALB) levels were estimated (Roche Hitachi 912), Chemistry Analyzer with Roche biochemical Kits. All tests were calibrated on two point and full calibration, as and when required, Precinorm and Precipath quality control serum were processed with each batch. Serum testosterone levels were estimated using Testosterone ELISA (RE52151) kit (Immuno Biological Laboratories, USA).

### Body and organ weight

Body weight, weight of testis, epididymis, seminal vesicles and ventral prostates, excised free of adhering tissues, were evaluated periodically every 20^th^,40^th^,60^th^ days during the treatment and 30^th^ day of recovery period.

### Sperm analysis

The cauda epididymis, wherein the spermatozoa mature and are stored, were dissected in 1000 μl of normal saline, and the clear fluid was used for the analysis of sperm concentration, motility, viability and abnormality, as per the procedures described in the WHO Laboratory Manual [17]. Acrosomal integrity is measured using fluorescently labeled plant lectins as described in WHO Laboratory Manual with modification [17]. Briefly acrosomal integrity of each sperm samples was smeared on glass slides and air-dried followed by fixation with 99% methanol. For staining, slides were incubated with (PSA–FITC, L0770, Sigma–Aldrich), at 37°C for 30 minutes, then washed with phosphate buffer saline and analyzed under microscope (Leica DMR fluorescence microscope) using an appropriate filter. The stained rat spermatozoa were classified into two groups; spermatozoa displaying intensively bright fluorescence in the acrosomal region were considered as intact acrosome and spermatozoa displaying weak, patchy, or no fluorescence in the acrosomal region were considered as damaged acrosome.

### Libido and fertility tests

Libido and fertility were assessed using the procedure as described previously [2]. Briefly at the termination of treatment schedule, three male rats from each group (randomly selected) were paired individually with two proven fertile females in estrous phase. Success of mating was confirmed by the presence of spermatozoa in the vaginal smear of the mated rats. The females were laparotomized on the 10^th^ day of postcoitum, and the number of corpus luteum implantation were counted if any, and number of litter deliver are recorded. Percentage preimplantation, post implantation loss and antifertility activity.

### Histological evaluation

The testis and other vital organ were used for histological studies at different stages of treatment and after recovery. For histology evaluation, testis were fixed in Bouin's fixative and other tissues in 10% formalin. Tissues were processed for wax embedding and embedded paraffin wax blocks were sectioned 5 μm thick and stained with haematoxylin and eosin.

### Statistical analysis

Analysis of each data set was performed by student-*t* test, and one-way analysis of variance (ANOVA). Statistically significant effects were further evaluated with Newman-Keuls tests. Differences were considered significant at P < 0.01. Results were expressed as means ± SEM.

## Results

### Safety evaluation

#### Evaluation of hematological and biochemical parameters in blood

No significant changes were observed in biochemical and hematological parameters when compared to control in SD, G I, G II, and G III rats. All hematological parameters were in normal ranges in the entire set of animal (results not shown). However, there was a significant reduction in serum cholesterol levels in G I, G II and G III group animals. Standard drug treatment significantly elevated the serum UA, SGOT, SGPT and ALP levels (Table 1).

**Table 1:** The biochemical parameters in rats treated orally with the Methanolic extract of the bark of *Aegle marmelos *(L.) 200 mg, 400 mg and 600 mg per kg body weight (bw) per day (values are mean ± SEM, n = six animals)

**Treatment schedule**		**Uric acid**	**SGOT**	**SGPT**	**ALK phosphate**	**Total protein**	**Albumin**	**Creatinine**	**Blood urea nitrogen**	**Cholesterol**
		**mg/dl**	**IU/L**	**IU/L**	**IU/L**	**gm/dl**	**gm/dl**	**mg/dl**	**mg/dl**	**mg/dl**
C		5.05 ± 1.20	26.83 ± 2.54	27.67 ± 2.64	70.07 ± 7.20	6.86 ± 1.30	3.95 ± 1.46	0.93 ± 0.15	14.47 ± 2.84	148.32 ± 12.2
SD	20 D	7.23 ± 2.51 ^a^	49.09 ± 4.87 ^a^	38.67 ± 3.54 ^a^	97.69 ± 10.47 ^a^	6.87 ± 1.27	3.54 ± 1.34	1.18 ± 0.31	15.60 ± 2.97	149.25 ± 12.4
	40 D	7.83 ± 2.57 ^a^	61.17 ± 3.86 ^a^	41.33 ± 4.08 ^a^	105.86 ± 12.49 ^a^	6.53 ± 1.17	3.26 ± 1.29	1.19 ± 0.32	16.69 ± 2.99	155.37 ± 13.74
	60 D	8.46 ± 2.79 ^a^	73.83 ± 4.19 ^a^	47.17 ± 4.56 ^a^	115.83 ± 13.41 ^a^	6.42 ± 1.04	3.22 ± 1.27	1.24 ± 0.37 ^a^	18.90 ± 3.14	160.5 ± 14.12
G I	20 D	5.14 ± 1.40	28.33 ± 1.11	26.17 ± 2.65	71.42 ± 7.24	6.96 ± 1.27	3.91 ± 1.45	0.88 ± 0.15	12.27 ± 2.17	138.79 ± 10.54
	40 D	5.017 ± 1.31	29.08 ± 2.87	27.5 ± 2.64	68.85 ± 7.01	7.21 ± 1.47	3.85 ± 1.43	0.940 ± 0.13	10.65 ± 1.94	135.24 ± 9.87
	60 D	5.65 ± 1.97	25.5 ± 2.43	22.5 ± 2.14	60.68 ± 6.50	7.11 ± 1.40	3.81 ± 1.40	0.91 ± 0.12	12.03 ± 2.07	124.33 ± 8.47 ^a^
G II	20 D	5.08 ± 1.31	27.17 ± 2.68	23.0 ± 2.20	57.87 ± 6.40	7.17 ± 1.88	3.86 ± 1.43	0.96 ± 0.1.8	11.81 ± 1.95	123.88 ± 8.44 ^a^
	40 D	6.94 ± 2.46	24.5 ± 2.37	23.33 ± 2.21	57.92 ± 6.42	7.26 ± 1.44	3.78 ± 1.40	0.96 ± 0.17	12.65 ± 2.23	118.79 ± 7.97 ^a^
	60 D	5.23 ± 1.49	30.0 ± 3.01	24.5 ± 2.36	58.05 ± 6.14	7.18 ± 1.40	3.85 ± 1.42	0.99 ± 0.19	13.87 ± 2.54	115.35 ± 7.49 ^a^
G III	20 D	5.86 ± 2.10	22.33 ± 2.14	25.5 ± 2.49	53.82 ± 5.68	7.35 ± 1.50	3.96 ± 1.47	0.98 ± 0.19	10.44 ± 1.89	120.54 ± 8.40 ^a^
	40 D	5.13 ± 1.47	27.17 ± 2.64	22.17 ± 2.10	53.07 ± 5.61	7.61 ± 1.87	3.83 ± 1.41	0.95 ± 0.17	12.12 ± 2.09	115.31 ± 7.48 ^a^
	60 D	5.017 ± 1.30	28.83 ± 2.76	21.33 ± 2.08	54.28 ± 5.84	7.08 ± 1.35	3.98 ± 1.49	0.91 ± 0.13	10.08 ± 1.74	110.23 ± 7.80 ^a^
RG I	30 D	5.47 ± 1.87	24.67 ± 2.38	23.0 ± 2.24	59.44 ± 6.04	7.25 ± 1.42	3.883 ± 1.42	0.98 ± 0.19	11.04 ± 1.90	136.48 ± 10.21
RG II	30 D	4.78 ± 1.19	28.04 ± 2.79	22.17 ± 2.17	58.21 ± 5.91	7.133 ± 1.39	3.917 ± 1.47	0.95 ± 0.17	11.22 ± 1.92	138.17 ± 10.43
RG III	30 D	4.71 ± 1.13	30.17 ± 3.09	24.17 ± 2.34	56.44 ± 5.74	7.037 ± 1.24	3.93 ± 1.47	0.98 ± 0.18	10.04 ± 1.70	143.2 ± 11.51

Significance level: ^a^ P ≤ 0.01 compared Control.

C- Rats treated with treated with in 2.0 ml 0.5 percent CMC each day for 60 days.

G I- Rats treated with methanolic bark extract of *Aegle marmelos* 200 mg/rat/day/p.o.

GII- Rats treated with methanolic bark extract of *Aegle marmelos* 400 mg/rat/day/p.o.

GIII- Rats treated with methanolic bark extract of *Aegle marmelos* 600 mg/rat/day/p.o.

RG-I- Rats withdraw from treated for 30 days after 60 days treatment of methanolic bark extract of *Aegle marmelos* 200 mg/rat/day/p.o.

RG-II- Rats withdraw from treated for 30 days after 60 days treatment of methanolic bark extract of *Aegle marmelos* 400 mg/rat/day/p.o.

RG-III- Rats withdraw from treated for 30 days after 60 days treatment of methanolic bark extract of *Aegle marmelos* 600 mg/rat/day/p.o.

### Evaluation of body vital organ and reproductive organ weights

The mean weights of the vital organs like brain, thyroid, heart, lungs, liver, spleen, adrenal gland and kidney did not show any significant differences at *P* < 0.05 throughout the treatment and 30 days after withdrawal of drug in recovery phase rats when compared to control group animals (Table 2). However, there is significant reduction in reproductive organs weight in standard drug and treated group animal in comparison to control animals at *P* < 0.01. Animal after removal from treatment for 30 days showed no significant difference in comparison to control groups at *P* < 0.01 except RG III (Table 3).

**Table 2:** The weight of vital organs in rats treated orally with the Methanolic extract of the bark of *Aegle marmelos *(L.) 200 mg, 400 mg and 600 mg per kg body weight (bw) per day (values are mean ± SEM, n = six animals)

**Treatment schedule**		**Brain**	**Thyroid**	**Heart**	**Lungs**	**Liver**	**Spleen**	**Adrenal**	**Kidney**
		**(g)**	**(g)**	**(g)**	**(g)**	**(g)**	**(g)**	**(g)**	**(g)**
C		1.828 ± 0.220	0.020 ±0.004	0.716 ±0.024	1.497 ±0.292	8.167 ±0.818	0.601 ±0.200	0.176 ± 0.002	1.742 ±0.240
SD	20 D	1.833 ± 0.243	0.020 ±0.004	0.714 ±0.024	1.502 ±0.301	8.0 ±0.801	0.614 ±0.210	0.177 ±0.002	1.765 ±0.261
	40 D	1.845 ± 0.254	0.020 ±0.004	0.741 ±0.028	1.487 ±0.281	7.967 ±0.793	0.625 ±0.220	0.175 ±0.002	1.752 ±0.251
	60 D	1.845 ± 0.250	0.020 ±0.004	0.726 ±0.027	1.508 ±0.301	7.967 ±0.791	0.615 ±0.211	0.178 ±0.002	1.755 ±0.252
G I	20 D	1.773 ± 0.194	0.019 ±0.003	0.681 ±0.194	1.457 ±0.251	8.307 ±0.830	0.611 ±0.210	0.176 ±0.002	1.743 ±0.241
	40 D	1.882 ± 0.276	0.019 ±0.003	0.724 ±0.027	1.519 ±0.312	8.213 ±0.820	0.593 ±0.197	0.173 ±0.002	1.728 ±0.227
	60 D	1.803 ± 0.204	0.020 ±0.004	0.720 ±0.026	1.498 ±0.300	7.945 ±0.790	0.616 ±0.211	0.178 ±0.002	1.720 ±0.220
G II	20 D	1.738 ± 0.187	0.020 ±0.004	0.723 ±0.026	1.517 ±0.311	8.249 ±0.820	0.592 ±0.196	0.177 ± 0.002	1.732 ±0.231
	40 D	1.858 ± 0.256	0.020 ±0.003	0.761 ±0.029	1.542 ±0.331	7.95 ±0.800	0.615 ±0.211	0.171 ±0.002	1.782 ±0.281
	60 D	1.82 ± 0.225	0.020 ±0.004	0.735 ±0.028	1.478 ±0.271	8.25 ±0.822	0.591 ±0.196	0.169 ±0.001	1.707 ±0.279
G III	20 D	1.842 ± 0.254	0.020 ±0.004	0.718 ±0.024	1.485 ±0.282	8.417 ±0.842	0.646 ±0.222	0.181 ±0.003	1.805 ±0.306
	40 D	1.728 ± 0.179	0.019 ±0.003	0.716 ±0.025	1.503 ±0.301	7.783 ±0.748	0.651 ±0.230	0.177 ±0.002	1.70 ±0.201
	60 D	1.898 ± 0.284	0.021 ±0.005	0.85 ±0.032	1.517 ±0.311	7.9 ±0.798	0.617 ±0.213	0.175 ±0.002	1.807 ±0.309
R G I	30 D	1.833 ± 0.243	0.020 ±0.003	0.733 ±0.027	1.518 ±0.313	8.1 ±0.810	0.594 ±0.198	0.179 ±0.002	1.79 ±0.280
R G II	30 D	1.828 ± 0.224	0.020 ±0.004	0.716 ±0.024	1.497 ±0.300	8.167 ±0.818	0.600 ±0.200	0.176 ±0.002	1.742 ±0.241
R G III	30 D	1.865 ± 0.255	0.020 ±0.003	0.810 ±0.031	1.493 ±0.300	8.051 ±0.806	0.594 ±0.194	0.175 ±0.002	1.803 ±0.307

Significance level: ^a^ P ≤ 0.01 compared Control.

C- Rats treated with treated with in 2.0 ml 0.5 percent CMC each day for 60 days.

SD- Rats treated with standard drug 50 mg/rat/day/p.o lonidamine.

G I- Rats treated with methanolic bark extract of *Aegle marmelos* 200 mg/rat/day/p.o.

GII- Rats treated with methanolic bark extract of *Aegle marmelos* 400 mg/rat/day/p.o.

GIII- Rats treated with methanolic bark extract of *Aegle marmelos* 600 mg/rat/day/p.o.

RG-I- Rats withdraw from treated for 30 days after 60 days treatment of methanolic bark extract of *Aegle marmelos* 200 mg/rat/day/p.o.

RG-II- Rats withdraw from treated for 30 days after 60 days treatment of methanolic bark extract of *Aegle marmelos* 400 mg/rat/day/p.o.

RG-III- Rats withdraw from treated for 30 days after 60 days treatment of methanolic bark extract of *Aegle marmelos* 600 mg/rat/day/p.o.

**Table 3:** The body weight and weight of reproductive organs in rats treated orally with the Methanolic extract of the bark of *Aegle marmelos *(L.) 200 mg, 400 mg and 600 mg per kg body weight (bw) per day (values are mean ± SEM, n = six animals)

**Treatment schedule**		**Body weight**	**Testis**	**Epididymis**	**Seminal vesicle**	**Ventral prostate**
		**(g)**	**(mg per 100 g bw)**	**(mg per 100 g bw)**	**(mg per 100 g bw)**	**(mg per 100 g bw)**
C		215.8 ± 3.47	829.2 ± 20.54	305.8 ± 14.18	510.0 ± 17.45	241.7 ± 9.80
SD	20 D	211.7 ± 2.90	775.0 ± 18.87 ^a^	298.3 ± 13.86	488.3 ± 16.33	213.3 ± 9.14
	40 D	210.8 ± 2.59	641.7 ± 15.49 ^a^	275.8 ± 13.67 ^a^	367.5 ± 13.10 ^a^	189.2 ± 7.92 ^a^
	60 D	208.3 ± 2.30	550.8 ± 13.16 ^a^	255.8 ± 12.88 ^a^	249.2 ± 9.34 ^a^	169.3 ± 6.14 ^a^
G I	20 D	213.3 ± 3.21	818.3 ± 19.93	309.2 ± 14.39	506.0 ± 17.32	232.5 ± 9.74
	40 D	214.2 ± 3.29	786.7 ± 18.85 ^a^	289.2 ± 13.21 ^a^	479.2 ± 16.42 ^a^	212.5 ± 9.10
	60 D	216.5 ± 3.57	730.8 ± 17.92 ^a^	264.2 ± 13.49 ^a^	436.7 ± 15.81 ^a^	201.5 ± 8.49 ^a^
G II	20 D	209.0 ± 2.43	800.8 ± 19.75 ^a^	307.5 ± 14.31	477.5 ± 16.28 ^a^	226.7 ± 9.48
	40 D	211.2 ± 2.84	754.2 ± 18.84 ^a^	278.3 ± 13.79 ^a^	383.3 ± 13.34 ^a^	205.0 ± 8.74
	60 D	208.3 ± 2.24	706.7 ± 17.56 ^a^	209.2 ± 11.90 ^a^	379.2 ± 13.17 ^a^	191.7 ± 8.00 ^a^
G III	20 D	216.2 ± 3.42	755.0 ± 18.8 ^a^	307.5 ± 14.34	399.2 ± 13.40 ^a^	208.8 ± 8.94
	40 D	208.7 ± 2.34	679.2 ± 15.95 ^a^	255.7 ± 12.30 ^a^	350.0 ± 11.93 ^a^	195.0 ± 8.25 ^a^
	60 D	216.3 ± 3.45	588.3 ± 13.71 ^a^	178.3 ± 10.02 ^a^	379.2 ± 13.20 ^a^	182.3 ± 7.87 ^a^
R G I	30 D	204.0 ± 1.85	812.3 ± 20.07	308.0 ± 14.39	503.3 ± 17.19	240.0 ± 9.84
R G II	30 D	214.7 ± 3.28	801.8 ± 19.75	300.8 ± 13.93	502.5 ± 17.05	226.7 ± 9.43
R G III	30 D	217.0 ± 3.63	792.5 ± 19.16 ^a^	298.2 ± 13.82	490.0 ± 16.87	206.2 ± 8.94

Significance level: ^a^ P ≤ 0.01 compared Control.

C- Rats treated with treated with in 2.0 ml 0.5 percent CMC each day for 60 days.

SD- Rats treated with standard drug 50 mg/rat/day/p.o lonidamine.

G I- Rats treated with methanolic bark extract of *Aegle marmelos* 200 mg/rat/day/p.o.

GII- Rats treated with methanolic bark extract of *Aegle marmelos* 400 mg/rat/day/p.o.

GIII- Rats treated with methanolic bark extract of *Aegle marmelos* 600 mg/rat/day/p.o.

RG-I- Rats withdraw from treated for 30 days after 60 days treatment of methanolic bark extract of *Aegle marmelos* 200 mg/rat/day/p.o.

RG-II- Rats withdraw from treated for 30 days after 60 days treatment of methanolic bark extract of *Aegle marmelos* 400 mg/rat/day/p.o.

RG-III- Rats withdraw from treated for 30 days after 60 days treatment of methanolic bark extract of *Aegle marmelos* 600 mg/rat/day/p.o.

### Serum testosterone levels

**Figure 1:**
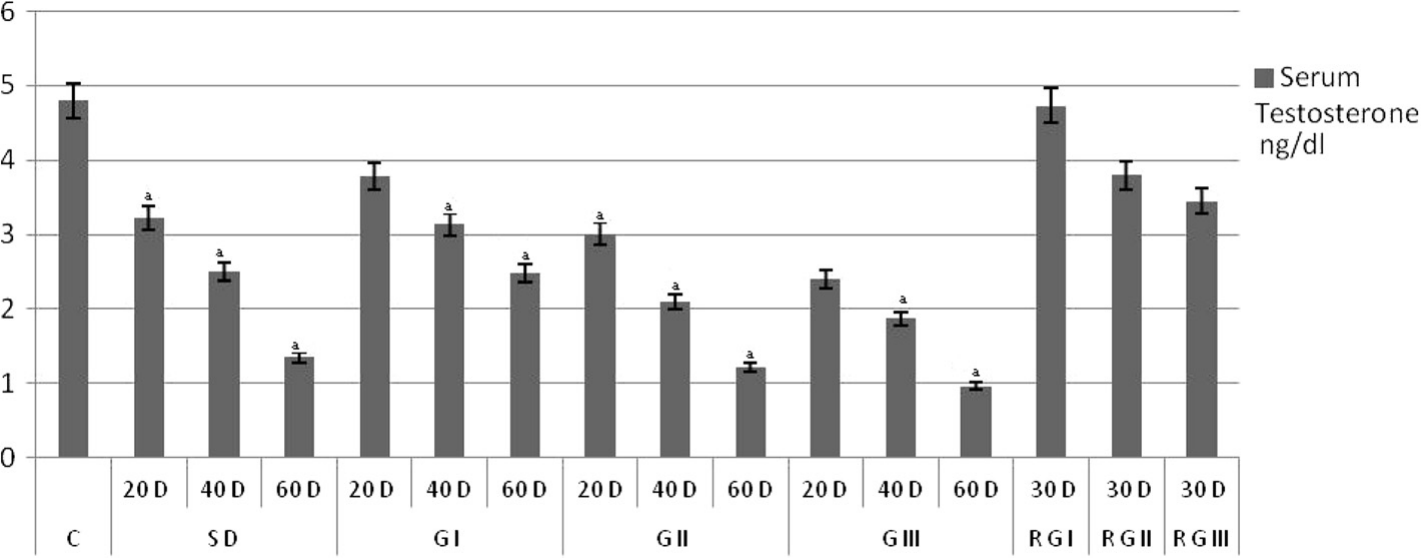
The Testosterone in rats treated orally with the Methanolic extract of the bark of Aegle *marmelos *(L.) 200 mg, 400 mg and 600 mg per kg body weight (bw) per day (values are mean ± SEM, n = six animals).

*Aegle marmelos* barks extract showed a dose and duration dependent decline in serum testosterone levels. SD and treatment groups G I, G II, and G III shown in marked reduction in testosterone levels compare to control group. Animals withdrawal from treatment for 30 days shows marked recovery in serum testosterone levels compare to control animals (Figure 1).

### Sperm concentration and motility

Sperm concentrations and motility in SD,G I, G II, and G III rats were significantly reduced at *P* < 0.01 compared to control on dose as well as time duration dependent Table 4. Where there was no sign of sperm motility in Standard drug G I and G II after 60^th^ days of treatment similarly, G II after 40^th^ and 60^th^ day’s treatment showed no sign of motility. Animal’s withdrawal from treatment for 30 days shows marked recovery compare to 60 days treated rats.

**Table 4:** The Cauda epididymal sperm characteristics and Preimplantation loss postimplantation loss and antifertility activity in rats control and treated orally with the Methanolic extract of the bark of *Aegle marmelos *(L.) 200 mg, 400 mg and 600 mg per kg body weight (bw) per day (values are mean ± SEM, n = six animals)

**Treatment schedule**		**Sperm density (million/mL)**	**Acrosomal integrity (%)**	**Sperm viability (%)**	**Sperm motility (%)**	**Pre implantation loss (%)**	**Post implantation loss (%)**	**Anti-fertility (%)**
C		39.47 ± 3.87	73.54 ± 7.21	67.83 ±5.67	77.17 ±7.60	14.36 ± 2.41	2.57 ± 1.60	18.32 ± 2.74
SD	20 D	28.92 ± 2.71 ^a^	57.17 ± 5.61 ^a^	56.67 ±4.74 ^a^	59.33 ± 6.01 ^a^	42.71 ± 6.41 ^a^	12.22 ± 2.94 ^a^	54.74 ± 8.42 ^a^
	40 D	21.27 ± 2.09 ^a^	49.00 ± 4.70 ^a^	46.07 ±3.63 ^a^	27.54 ± 2.84 ^a^	60.05 ± 9.04 ^a^	6.43 ± 0.79 ^a^	66.48 ± 9.64 ^a^
	60 D	16.77 ±1.79 ^a^	38.17 ± 3.71 ^a^	27.04 ± 3.01 ^a^	NIL	91.21 ± 14.14 ^a^	8.79 ± 1.1 ^a^	100.0 ± 0 ^a^
G I	20 D	34.96 ±3.40	64.83 ± 6.40 ^a^	61.17 ± 5.20 ^a^	61.33 ±6.09 ^a^	23.32 ± 3.84 ^a^	18.22 ± 3.20 ^a^	41.54 ± 6.17 ^a^
	40 D	28.65 ±2.76 ^a^	56.67 ± 5.59 ^a^	54.17 ±4.34 ^a^	30.17 ±3.05 ^a^	45.57 ± 6.71 ^a^	14.62 ± 3.07 ^a^	60.19 ± 9.01 ^a^
	60 D	21.48 ±2.10 ^a^	41.59 ± 4.10 ^a^	44.17 ±3.57 ^a^	3.67 ±3.71 ^a^	100.0 ± 0 ^a^	NA	100.0 ± 0 ^a^
G II	20 D	26.22 ±2.51 ^a^	52.17 ± 5.21 ^a^	52.00 ±4.20 ^a^	50.83 ± 5.09 ^a^	41.35 ± 6.21 ^a^	16.89 ± 3.14 ^a^	58.23 ± 8.76 ^a^
	40 D	20.92 ±1.98 ^a^	41.17 ± 4.09 ^a^	45.56 ±3.61 ^a^	27.33 ± 2.81 ^a^	63.03 ± 9.18 ^a^	21.94 ± 3.71 ^a^	84.97 ± 11.43 ^a^
	60 D	16.88 ±1.54 ^a^	16.17 ± 1.71 ^a^	38.67 ±3.12 ^a^	Nil	100.0 ± 0 ^a^	NA	100.0 ± 0 ^a^
G III	20 D	26.0 ±2.51 ^a^	40.67 ± 4.01 ^a^	48.33 ±3.84 ^a^	38.50 ±3.91 ^a^	57.38 ± 8.79 ^a^	6.91 ± 2.19 ^a^	64.34 ± 9.45 ^a^
	40 D	17.05 ±1.67 ^a^	15.07 ± 1.54 ^a^	34.83 ±2.97 ^a^	Nil	100.0 ± 0 ^a^	NA	100.0 ± 0 ^a^
	60 D	12.33 ±1.27 ^a^	9.833 ± 0.94 ^a^	21.17 ±2.21 ^a^	Nil	100.0 ± 0 ^a^	NA	100.0 ± 0 ^a^
RG I	30 D	37.14 ±3.67	62.83 ± 6.21 ^a^	63.0 ±5.50	72.33 ±7.34	16.25 ± 2.74	2.57 ± 1.59	18.82 ± 2.87
RG II	30 D	35.52 ±3.44	55.00 ± 5.49 ^a^	63.83 ±5.67	64.83 ±6.50	19.77 ± 1.57	2.21 ± 1.40	21.99 ± 3.90
RG III	30 D	30.33 ±2.97	49.67 ± 4.87 ^a^	57.17 ±4.74	63.83 ±6.44	23.14 ± 3.77	2.89 ± 1.71	26.04 ± 4.10

Significance level: ^a^ P ≤ 0.01 compared Control.

C- Rats treated with treated with in 2.0 ml 0.5 percent CMC each day for 60 days.

G I- Rats treated with methanolic bark extract of *Aegle marmelos* 200 mg/rat/day/p.o.

GII- Rats treated with methanolic bark extract of *Aegle marmelos* 400 mg/rat/day/p.o.

GIII- Rats treated with methanolic bark extract of *Aegle marmelos* 600 mg/rat/day/p.o.

RG-I- Rats withdraw from treated for 30 days after 60 days treatment of methanolic bark extract of *Aegle marmelos* 200 mg/rat/day/p.o.

RG-II- Rats withdraw from treated for 30 days after 60 days treatment of methanolic bark extract of *Aegle marmelos* 400 mg/rat/day/p.o.

RG-III- Rats withdraw from treated for 30 days after 60 days treatment of methanolic bark extract of *Aegle marmelos* 600 mg/rat/day/p.o.

### Sperm viability

Treatment with standard drug and *Aegle marmelos* barks extract caused a significant decrease P < 0.01 in the viable spermatozoa compare to control animals. On withdrawal of the treatment for 30 days shows marked restoration of sperm viability compare to 60 days of treatment and no significant difference compare to Control group animals (Table 4).

### Acrosomal integrity

*Aegle marmelos* bark extract treated group showed dose as well as duration dependent defect in acrosomal integrity. Sperms from G I showed decline in acrosomal integrity compare to control animals 88.15%, 77.06%, 56.55% on 20^th^, 40th and 60^th^ day respectively. Similarly in G I and GII acrosomal integrity was reduced to 70.94% & 55.30% after 20^th^ day treatment 55.93% & 20.49% after 40^th^ day of treatment, 21.98 & 13.37% after 60^th^ day of treatment when compare to control group. A significant revival of acrosomal integrity was observed after withdrawal from treatment for 30 days 85.43%, 74.78% and 67.57% in RG I, RG II and RG III groups respectively when compare to control group (Table 4).

### Libido and fertility tests

A dose and duration dependent effect was observed in the fertility test for the treated males mated with normal fertile females. Libido of the treated animals was unaffected. Complete inhibition of fertility in terms of offspring deliveries of the female were observed at 60 days treatment in SD, G I. and G II. In case of G III 100% antifertility activity appears from 40 days onwards. A significant restoration in fertility which was equal comparable to controls after 30 days withdrawal from treatment resulted as shown in (Table 4).

### Histology of the testis

**Figure 2:**
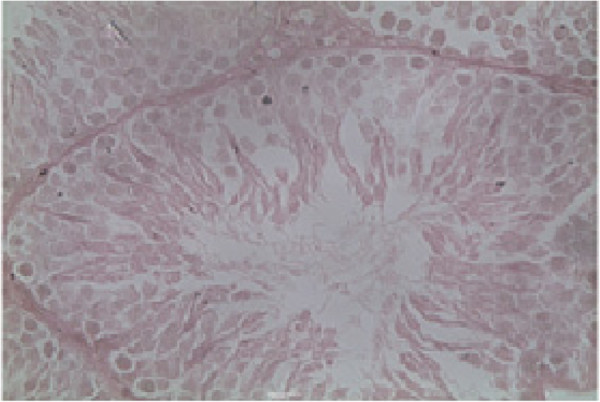
**Histology of the testis of a control animal.** The seminiferous tubules contain sertoli cells and germ cells of various stages, covering the entire process of spermatogenesis. The lumen contains mature spermatozoa. The interstitium contains distinct leydig cells, scale bar = 10 μm.

**Figure 3:**
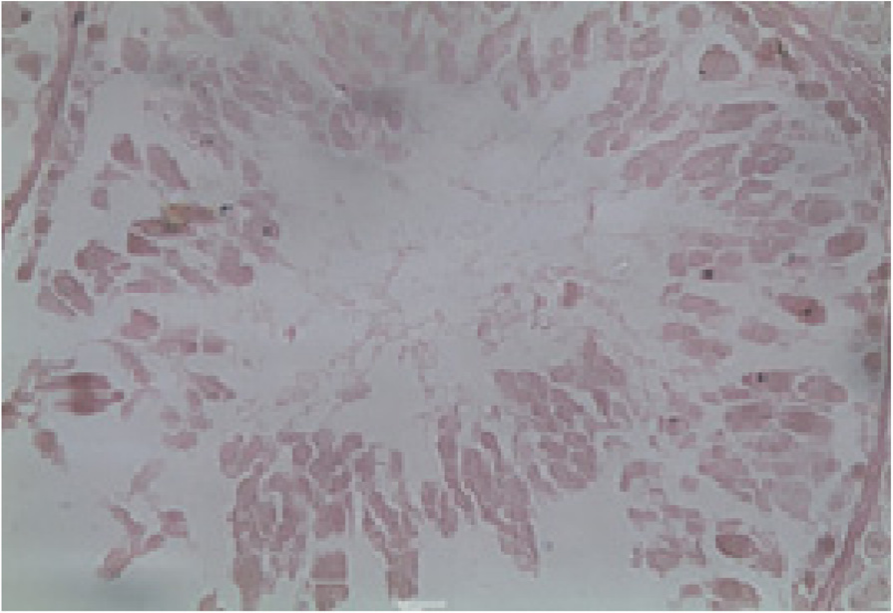
**Histology of the methanolic extract of the bark of *Aegle marmelos *(L.) at a dose level 400 mg/kg b.w for 40 days orally treated rat.** Shows structural disorganization and mild to modrate exfoliation of elongated spermatids was apparent in some tubules. Large multinucleated cells were also present in a number of tubules, scale bar = 10 μm.

**Figure 4:**
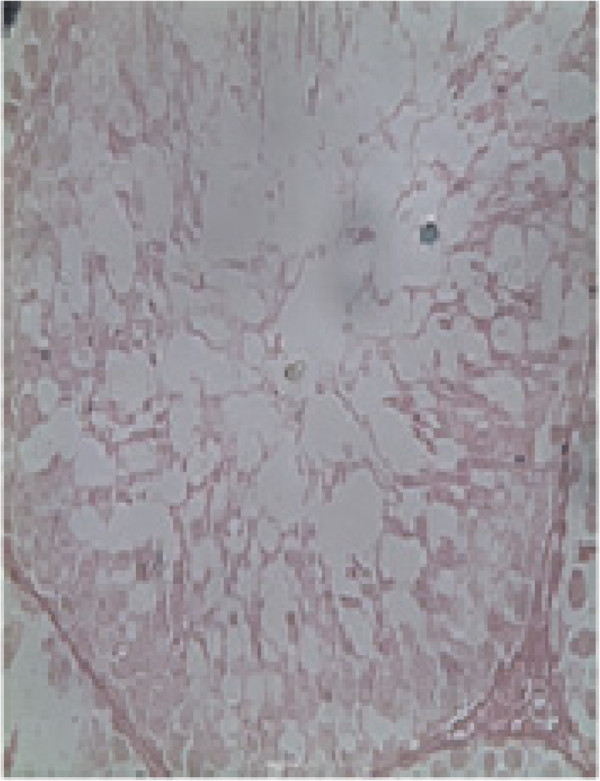
**Histology of the methanolic extract of the bark of *Aegle marmelos *(L.) at a dose level 400 mg/kg b.w for 40 days orally.** sertoli cells show vacuolization, whereas the spermatogonia, spermatocytes and spermatids (round as well as elongated) appear normal, with normal nuclear and cytoplasmic characteristics, scale bar = 10 μm.

**Figure 5:**
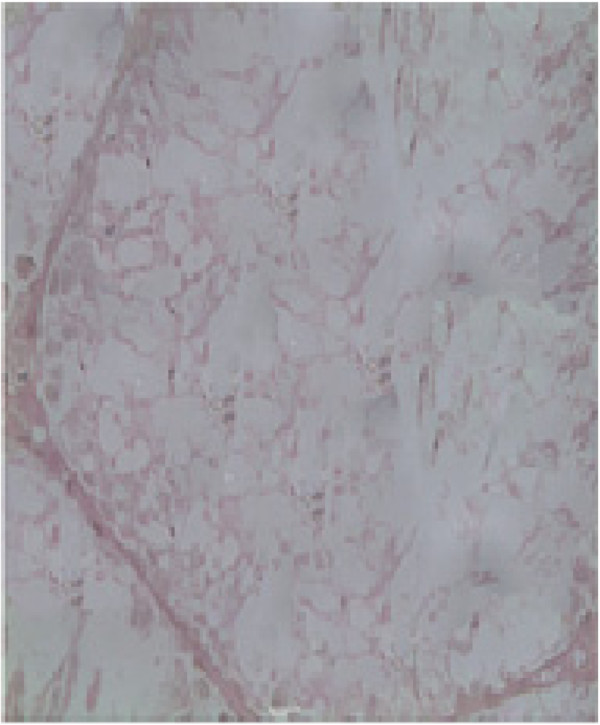
**Histology of the methanolic extract of the bark of *Aegle marmelos *(L.) at a dose level 400 mg/Kg b.w for 60 days orally**. The disruption of spermatogenesis is evident. Vacuolization is evident in the cytoplasm of sertoli cells, spermatocytes and spermatids. The nuclei of spermatocytes and spermatids show pyknosis, scale bar = 10 μm.

**Figure 6:**
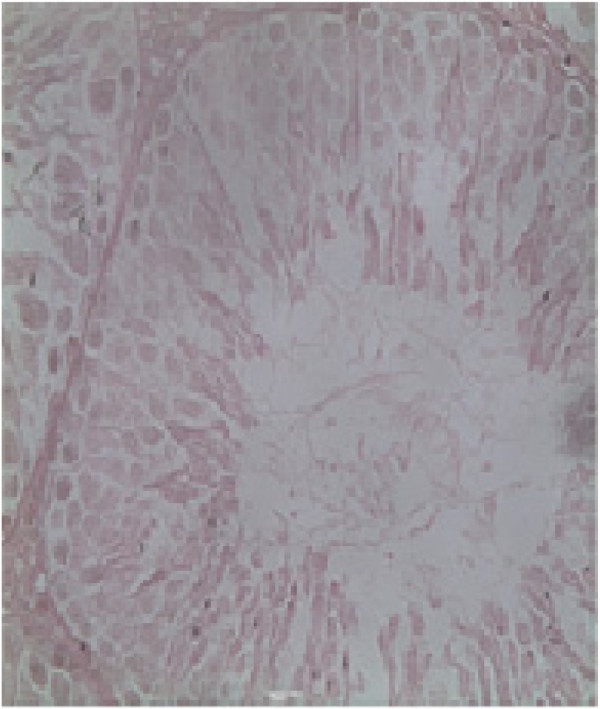
**Rats withdraw from treated for 30 days after 60 days treatment of methanolic bark extract of *Aegle marmelos *400 mg orally with the methanolic extract of the bark of *Aegle marmelos *(L.) Restoration of spermatogenesis is evident.** The seminiferous epithelium is comparable with that of a control animal. The sertoli cells and germ cells show closer association to each other, with normal nuclear and cytoplasmic characteristics. The lumen contains packed spermatozoa, and the leydig cells appear normal, scale bar = 10 μm.

Histopathological examination of the testis at specified intervals throughout the treatment period was performed. Histopathological examination of testes in control animal showed seminiferous tubules of the testes possessed epithelia containing the sertoli cells and the germ cells at various stages, covering the complete process of spermatogenesis. Sertoli cells exhibited typical, irregular nuclei and well-defined cytoplasm, which appeared granular. The spermatogonia, oval in shape, were closely associated with the basal lamina. Spermatocytes showed various degrees of condensation of their nuclei and were closely associated with sertoli cell cytoplasm. The lumen contained mature spermatozoa, and the interstitium contained distinct Leydig cells (Figure 2). Group SD, G I, G II and G III animals showed dose and time duration dependent defect on histopathology of testes as in G I on 20^th^ and 40^th^ days and G II at 20^th^ days treatment shows mild to moderate exfoliation of elongated spermatids was apparent in some tubules. Large multinucleated cells were also present in a number of tubules (Figure 3). In addition, prominent spaces detected within the germinal epithelium were consistent with structural disorganization. Group II on 40^th^ day and Group III at 20^th^ and 40^th^ days testes show nuclear chromatin condensation and degeneration (Figure 4). The cytoplasm of all germ cells shows vacuoles and tubules suggesting degeneration of their germinal epithelium. Many tubules showed signs of restructuring lumen contained reduced spermatozoa, and the Leydig cells. Disruption of spermatogenesis were evident (Figure 5) after treatment of 60 days in G II and G III. After 30 days of recovery, restoration of proper spermatogenesis was evident. The seminiferous epithelium was comparable with that of control animals. The sertoli cells and germ cells showed closer associations, with normal nuclear and cytoplasmic characteristics (Figure 6).

## Discussion

*Aegle marmelos* barks extract on oral administration to male rats showed an antifertility activity without any apparent effects on organs other than those involved in the reproductive process. A dose and time duration dependent depletion of the germinal epithelium, reduction in serum testosterone and eventual return of spermatogenesis and testosterone levels are indicative of the potential of this extract as a male contraceptive with reversibility. In addition, the extract reduces the quality of sperm in a dose and time dependent manner. *Aegle marmelos* barks extract on oral administration of 200 & 400 mg/kg b.w produce 100% antifertility effect on 60^th^ day and 600 mg/kg b.w on 40^th^ days onwards. This delayed onset effect could be due to the epididymal capacity for sperm storage which is approximately 30 days or beyond, the sperm pool may have been resorbed instead of damaged [18]. Alterations in the sperm motility, viability and histopathological examination of testes suggested a disturbed testicular and epididymal microenvironment due to treatment as evidenced by significant reduction in the tissues weight. There was significant increase in number of vacuoles in extract treated group thereby reduction in the epithelial cell mass in the testes leading to the substantial loss of mass from the testes. Extract selectively affect the sex-organs either due to the some of the chemical compounds (marmin and fagarine) present in the extract which is selectively stored in the sex organs. It is well established that active sperm motility is a prerequisite to achieve fertilization. Sperms ejaculated into the vagina must reach to the ovum in the fallopian tube by penetrating the cervical mucus and passing through the uterine cavity. Because an adequate number of sperms possessing normal function are necessary for successful fertilization, any deviation that alters sperm function leads to infertility [19]. The results of this investigation show that dose as well time duration treatment with methanolic extract of *Aegle marmelos* bark for a period up to 60 days is safe and effective in completely inhibiting the sperm motility in rats. This inhibition coincides with a gradual and significant decline in cauda epididymal sperm density, percentage of viable spermatozoa, acrosomal integrity and a significant increase in sperm anomalies, degenerated germinal epithelium, vacuolization in sertoli cells and proliferating germ cells. Furthermore, disturbances in spermatid differentiation of the testis, reduction in serum testosterone levels and sterility indicate significant contraceptive efficacy. The onset of contraceptive efficacy is evident within 60 days of treatment in Group I & II and 40 days in Group III as confirmed by fertility tests. Preimplantation loss is major cause of infertility and *A*.*marmelos* methanolic extract mainly act during preimplantation stage. It is likely that any contraceptive agent that affects sperm motility would influence spermatozoa indirectly either through disruption of epididymal epithelial cell function or by acting directly on the spermatozoa by disrupting their enzymes [20]. Therefore it is likely that *Aegle marmelos* act by either of these mechanisms mentioned above. However *Aegle marmelos* barks extract showed a protective effect on lipids profile by increasing high density lipoprotein (HDL) levels and lipid profiles which are directly related to sperm motility and concentration [21]. Enzymes present on acrosome is of paramount importance for attachment and penetration of the sperm in ova, acrosomal integrity if compromised will adversely affect ability of sperm to fertilise the ovum. *Aegle marmelos* was found to reduce the acrosomal integrity in dose and time dependent manner. *Aegle marmelos* contains a coumarin derivative marmin which is known for selectively inhibiting the calcium channels [22]. Calcium channel blockade might be a vital mechanism through which *A.marmelos* extract is acting as spermicidal agent. Role of calcium channels is implicated in the sperm viability [23,24] as it mediate acrosomal reaction of human sperm during fertilization and inhibitors of these channels may prevent sperm–egg fusion [25,26]. This is further supported by reduction in the implants found during the fertility studies in proven fertile female rats.

Preimplantation loss due to *Aegle marmelos* methanolic extract suggests that the extract act on sperm parameters and produces contraception. It is interesting to observe that these compounds are found to be abundant in bark extract in comparison to leaf and other parts. Fagarine, a compound isolated from *Aegle marmelos* belongs to benzo phenanthridines alkaloids, are cytotoxic and anti-androgenic in nature, [27,28] it is quite possible that it may have anti-androgenic behavior which could lead to its spermicidal properties.

## Conclusions

Our research showed that oral administration of *Aegle marmelos* barks methanolic extract lead to a dose as well as time duration dependent defects in the testicular spermatogenesis which leads to production of defective sperms. Male rats with such defective sperms quality and quantity are unable to produce the offspring however the libido of male remains ineffective during this period. Our studies also showed that after widrawal administration of *Aegle marmelos* bark methanolic extract restores these defects. Our studies also suggest a strong candidature of *Aegle marmelos* barks methanolic extract for male contraceptive via its ability to produce complete inhibition of pregnancy, restoration fertility after withdrawal from treatment and its lipid correcting ability proven for further beneficial effect (data of HDL and TG not shown) and does not cause toxic effect on liver or kidney.

## Competing interests

The author(s) declare that they have no competing interests.

## Authors’ contributions

Agrawal SS performed overall supervision of the research project; Singh V, Gullaiya S carried out pharmacological and experimental research work; Kumar A, Dubey V carried out statistical, analysis and interpretation of data; Nagar A carried out the pharmacognostical studies; Tiwari P , Dhar P performed acquisition of data and manuscript drafting. All authors read and approved the final manuscript.
